# A novel, nested, multiplex, real-time PCR for detection of bacteria and fungi in blood

**DOI:** 10.1186/1471-2180-14-144

**Published:** 2014-06-04

**Authors:** Tomasz Gosiewski, Danuta Jurkiewicz-Badacz, Agnieszka Sroka, Monika Brzychczy-Włoch, Małgorzata Bulanda

**Affiliations:** 1 Department of Bacteriology, Microbial Ecology and Parasitology, Chair of Microbiology Jagiellonian University Medical College, Czysta 18 Str, 31-121 Kraków, Poland; 2 Department Epidemiology of Infection, Chair of Microbiology Jagiellonian University Medical College, Czysta 18 Str, 31-121 Kraków, Poland; 3 The John Paul II Hospital in Krakow, Pradnicka 80 Str, 31-202 Kraków, Poland

**Keywords:** Nested PCR, Multiplex PCR, Sepsis, Bacteria, Fungi, Gram differentiation

## Abstract

**Background:**

The study describes the application of the PCR method for the simultaneous detection of DNA of Gram-negative bacteria, Gram-positive bacteria, yeast fungi and filamentous fungi in blood and, thus, a whole range of microbial etiological agents that may cause sepsis. Material for the study was sterile blood inoculated with four species of microorganisms (*Escherichia coli*, *Staphylococcus aureus*, *Candida albicans* and *Aspergillus fumigatus*) and blood collected from patients with clinical symptoms of sepsis. The developed method is based on nested-multiplex real-time PCR .

**Results:**

Analysis of the obtained data shows that sensitivity of nested-multiplex real-time PCR remained at the level of 10^1^ CFU/ml for each of the four studied species of microorganisms and the percentage of positive results of the examined blood samples from the patients was 70% and 19% for the microbiological culture method. The designed primers correctly typed the studied species as belonging to the groups of Gram-positive bacteria, Gram-negative bacteria, yeast fungi, or filamentous fungi.

**Conclusions:**

Results obtained by us indicated that the designed PCR methods: (1) allow to detect bacteria in whole blood samples, (2) are much more sensitive than culture method, (3) allow differentiation of the main groups of microorganisms within a few hours.

## Background

Given the nonspecific clinical symptoms of sepsis, especially in its early stages, and the need for rapid implementation of appropriate therapy, microbiological and laboratory testing is of importance. The key role in diagnostics is determining the etiological agent of infection. Until now, the so-called diagnostic “gold standard” is still blood cultures performed in specialized media, preferably in automated culture systems. An important advantage of blood cultures is their low cost of testing. However, the long period of waiting for the results, in relation to the need for rapid implementation of appropriate antibiotic therapy, is undoubtedly a disadvantage of this method. The downside is also its low sensitivity – positive blood culture results, despite the presence of clinical signs of sepsis, are obtained in less than 50% of cases [1,2]. The situation is further exacerbated by subjecting patients to antibiotherapy before the collection of blood samples for culture – patients are often treated with antibiotics before the symptoms of sepsis manifest themselves. In such cases, cultures from blood are very difficult to perform due to the fact that it contains antibiotics inhibiting the growth of microorganisms. The detection of microbial nucleic acids is promising for effective, accurate and prompt diagnostics of bloodstream infections. The sensitivity of molecular methods is much higher than the sensitivity of the culture method, and, what is more, prior employment of antibiotherapy does not affect the test results [3]. Unfortunately, methods of molecular biology also have their limitations. The difficulty is caused by the isolation of DNA of suitable quality and high concentration from blood. An essential step in isolating DNA from blood is bacterial and fungal cell wall lysis of its cells present in the blood. However, bacteria and fungi display varying susceptibility to lysis. The wall of Gram-positive bacteria and fungi is thick and resistant to degradation, which results in the necessity of employing mechanical disruption, chemical lysis, and thermal lysis [4]. Another problem is the amplification of microbial DNA isolated from blood which can be inhibited by heme, its main component. This compound causes dissociation of DNA polymerase which results in disintegration of enzyme–substrate complex, and, additionally, it blocks its catalytic pocket [5-7]. The listed difficulties cause the market to lack solutions which have applications in molecular diagnostics of sepsis. Although it is possible to point out SeptiFast (Roche) kit which, however, does not exhaust the possibilities offered by the application of the diagnostic method based on PCR [8,9]. Septifast (Roche) allows the detection of ten to twenty species of bacteria and fungi, whose presence is most often confirmed in patients’ blood. However, if sepsis is triggered by a bacterial or fungal etiological agent from outside the list, the Septifast kit will generate a negative test result, which may mislead the physician.

Consequently, the aim of the study was to develop an alternative nested, multiplex, real time PCR (qPCR) method serving to detect the presence of microbes in blood in order to diagnose sepsis.

## Results

### Primers design

Four external primer sequences have been designed. Their sequences are presented in Table 1. A test of the designed primers was performed on DNA isolated from the reference strains of the bacterial and fungal species listed in the section “Reference microbial strains” and amplification signal was obtained for all species, with no cross-reaction of primers specific for bacteria to fungal DNA and vice versa. The designed primers correctly typed the studied reference strains species as belonging to the groups of Gram-positive bacteria, Gram-negative bacteria, yeast fungi, or filamentous fungi.

**Table 1:** Sequences of primers and probes utilized in the study

**Amplification**	**Oligonucleotide**	**Sequence 5′ - 3′**	**Origin**	**Target sequences**
**I**	*EXT_BAC_F	kGCGrACGGGTGAGTAA	in this study	16S rRNA
	*EXT_BAC_R	CGCATTTCACCGCTA	in this study	
	*EXT_FUN_F	AATTGACGGAAGGGCACC	in this study	18S rRNA
	*EXT_FUN_R	TTCCTCGTTGAAGAGCAA	in this study	
**II**	**FUN_F	TTGGTGGAGTGATTTGTCTGCT	[11]	18S rRNA
	**FUN_R	TCTAAGGGCATCACAGACCTG		
	Candid_probe	FAM-TTAACCTACTAAATAGTGCTGCTAGC-BHQ1		
	Asperg_probe	TexasRed-TCGGCCCTTAAATAGCCCGGTCCGC-Eclipse		
	**GN/GP_F	GACTCCTACGGGAGGC	[10]	16S rRNA
	**GN/GP_R	GCGGCTGCTGGCAC		
	GP_Probe	Hex- CTGAyssAGCAACGCCGCG –TAMRA (Q)		
	GN_Probe	Cy5 –CCTGAysCAGCmATGCCGCG- BHQ-2		
**β-actin gene**	F	GCCAGTGCCAGAAGAGCCAA	[16]	β-actin gene
	R	TTAGGGTTGCCCATAACAGC		

*External primers; **internal primers; k = G/T; r = A/G.

### The multiplex real-time PCR amplification standardization

The annealing temperature of the primers (amplification I) was determined to be 46.0°C and for amplification II – 65.0°C (Table 2). Afterwards, it was arranged that magnesium ion concentration should equal 6.5 mM for amplification I and 11.5 mM for amplification II. Compositions of the reaction mixtures were presented in Table 2. Concentration of the used reagents were as follows: external primers (Genomed) – 10 μM; internal primers (Genomed) – 20 μM; TaqMan probes (Genomed) – 20 μM; Buffer B 10× (EURx); dNTP’s (EURx) – 2 mM; MgCl_2_ (DNAGdansk) – 50 mM; Perpetual *Taq* Polymerase 2,5 U/μl (EURx). DNA amplification was carried out under the following thermal conditions for amplification I: 95°C for 5 min (95°C for 20 s, 46°C for 20 s, 72°C for 30 s) 30 cycles and for amplification II: 95°C for 5 min (95°C for 15 s, 65°C for 1 min) 40 cycles.

**Table 2:** The composition of the reaction mixtures, the reagents involved and PCR reaction thermal profiles

**NESTED multiplex qPCR**				**Multiplex qPCR**	
				**[final volume 50 μl]**	
**I amplification**		**II amplification**			
**[final volume 25 μl]**		**[final volume 10 μl]**			
1. H_2_O	6,7 μl	1. H_2_O	2,08 μl	1. H_2_O	0,4 μl
2. Buffer B	2,5 μl	2. Buffer B	1,0 μl	2. Buffer B	5,0 μl
3. EXT_BAC_F	0,125	3. GN/GP_F	0,2 μl	3. GN/GP_F	1,0 μl
4. EXT_BAC_R	0,125	4. GN/GP_R	0,2 μl	4. GN/GP_R	1,0 μl
5. EXT_FUN_F	0,125	5. GP_probe	0,05 μl	5. GP_probe	0,25 μl
6. EXT_FUN_R	0,125	6. GN_probe	0,05 μl	6. GN_probe	0,25 μl
7. dNTP’s	2,5	7. FUN_F	0,2 μl	7. FUN_F	1,0 μl
8. MgCl_2_	2,5	8. FUN_R	0,2 μl	8. FUN_R	1,0 μl
9. Polymerase Perpetual *Taq*	0,3	9. Asperg_prob	0,05 μl	9. Asperg_prob	0,25 μl
10. DNA	10	10. Candid_probe	0,05 μl	10. Candid_probe	0,25 μl
		11. dNTP’s	1,0 μl	11. dNTP’s	5,0 μl
		12. MgCl_2_	1,8 μl	12. MgCl_2_	9,0 μl
		13. Polymerase Perpetual *Taq*	0,12 μl	13. Polymerase Perpetual *Taq*	0,6 μl
		14. DNA (product of I amplification)	3,0 μl	14. DNA	25,0 μl

### Evaluation of the qPCR method sensitivity

The indication of sensitivity was performed separately for amplification II (internal primers) and in the nested system, i.e. in successive amplifications I and II. The obtained results were compared in Table 3. These results allow us to conclude that the use of amplification in the nested system, i.e. successive amplifications I and II, gives us the possibility to increase the detection sensitivity by two orders of magnitude for reference strains of filamentous, yeast fungi and for Gram-positive and Gram-negative bacteria in comparison with amplification II alone – functioning as an independent reaction.

**Table 3:** Comparison of sensitivity of the nested multiplex PCR and multiplex PCR methods in real time

**Microbe species**	**Order of magnitude [CFU/ml]**						**Detection limit of the multiplex qPCR**	**Order of magnitude [CFU/ml]**						**Detection limit of the nested multiplex qPCR [CFU/ml]**	*RFU level
	**10**^**5**^	**10**^**4**^	**10**^**3**^	**10**^**2**^	**10**^**1**^	**10**^**0**^	**[CFU/ml]**	**10**^**5**^	**10**^**4**^	**10**^**3**^	**10**^**2**^	**10**^**1**^	**10**^**0**^		
	+	+	+	∅	∅	∅		**+**	**+**	**+**	**+**	**+**	∅		**100**
***A. fumigatus***	+	+					**3.7 × 10**^**3**^ **± 2.4 × 10**^**3**^	**+**	**+**	**+**	**+**			**3.7 × 10**^**1**^ **± 2.7 × 10**^**2**^	
(filamentous fungi)	+	+					3.2 × 10^3^ CFU/reaction	**+**	**+**	**+**	**+**			1.2 CFU/reaction	
	+	+					**C_T_ (31.2-38.5)	**+**	**+**	**+**	**+**			C_T_ (29.1-32.2)	
	+	+						**+**	**+**	**+**	**+**				
	+	+	+		∅	∅		**+**	**+**	**+**	**+**		∅		**50**
***C. albicans***	+	+	+	+			**9.9 × 10**^**2**^ **± 3.4 × 10**^**3**^	**+**	**+**	**+**	**+**	**+**		**8.5 × 10**^**1**^ **± 3.6 × 10**^**2**^	
(yeast)	+	+	+	+			9.5 × 10^1^ CFU/reaction	**+**	**+**	**+**	**+**	**+**		0.24 CFU/reaction	
	+	+	+				C_T_ (33.3-37.2)	**+**	**+**	**+**	**+**	**+**		C_T_ (29.7-32.1)	
	+	+	+					**+**	**+**	**+**	**+**	**+**			
	+	+	+	∅	∅	∅		**+**	**+**	**+**	**+**	**+**	**+**		**100**
***S. aureus***	+	+	+				**4.5 × 10**^**3**^ **± 2.5 × 10**^**3**^	**+**	**+**	**+**	**+**	**+**		**1.1 × 10**^**1**^ **± 3.7 × 10**^**2**^	
(Gram positive bacteria)	+	+	+				5.1 × 10^2^ CFU/reaction	**+**	**+**	**+**	**+**	**+**		0.78 CFU/reaction	
	+	+	+				C_T_ (34.0-36.7)	**+**	**+**	**+**	**+**	**+**		C_T_ (25.2-28.0)	
	+	+						**+**	**+**	**+**	**+**	**+**			
	+	+		∅	∅	∅		**+**	**+**	**+**	**+**	**+**	**+**		**30**
***E. coli***	+	+	+				**5.4 × 10**^**3**^ **± 2.5 × 10**^**2**^	**+**	**+**	**+**	**+**	**+**		**1.3 × 10**^**1**^ **± 3.7 × 10**^**2**^	
(Gram negative bacteria)	+	+	+				6.1 × 10^2^ CFU/reaction	**+**	**+**	**+**	**+**	**+**		0.73 CFU/reaction	
	+	+	+				C_T_ (30.5-33.2)	**+**	**+**	**+**	**+**	**+**		C_T_ (24.4-27.2)	
	+	+	+					**+**	**+**	**+**	**+**	**+**			

“+”- the number of positive amplification results conducted in the five repetitions; “∅” – lack of amplification signal.

*RFU (relative fluorescence unit) signifies the value of the designated baseline.

**C_T_ value, i.e. the consecutive reaction cycle number in which the linear increase of the product cut the established baseline RFU level.

### The study of blood samples from patients using the method of nested-multiplex qPCR, multiplex qPCR and microbiological blood culture

102 blood samples from patients with clinical symptoms of sepsis were examined with the use of the developed method in the nested multiplex system and of BacT/ALERT® 3D (bioMérieux) blood culture. The application of the developed method for microbial detection allowed to increase the percentage of positive results from 18.6% as for culture to 69.6% in the case of nested-multiplex qPCR (Table 4). The elaborated PCR method enabled us to confirm the results of blood culture in every case and assign group membership, being Gram-positive bacteria or Gram-negative bacteria – yeast fungi presence was confirmed in one case only by PCR (the presence of filamentous fungi was not demonstrated). Mutliplex qPCR (no nested-PCR) gave results of 17.6% which is a value slightly lower than in the case of using culture methods and just as nested PCR confirm the results of blood culture. In all 102 samples, amplification signal in negative control was not obtained, which guarantees absence of contamination. A detailed compilation of the results is presented in Table 4.

**Table 4:** Comparison of the results obtained from blood of patients with clinical symptoms of sepsis by the method of blood culture, the nested multiplex qPCR and the multiplex qPCR methods in the parentheses and the internal inhibition control for the β-actin gene

	**Blood culture**	**Nested-multiplex-qPCR (n = 102)**				**Total positive samples**	**β-actin gene**
		**(Multiplex-qPCR)**					
	**(n = 102)**	**Gram positive**	**Gram negative**	**Yeast**	**Filamentous fungi**		**(n = 102)**
		43	51	1	0		
		(16)	(18)	(0)	(0)	71	
**Positive**	19					(18)	102
		42.1	50.0	1	0		
		(15.7)	(17.6)	(0)	(0)	69.6	
**%**	18.6					(17.6)	100.0

“( )” – in the parentheses Multiplex-qPCR results.

## Discussion

Molecular diagnostics of microbial etiological agents of sepsis is currently at an initial stage and is limited more to scientific research than to diagnostic practice. Only few kits for the detection of microorganisms that cause sepsis are available on the market: SeptiFast (Roche) and SeptiTest (Molzym), but in no way do they satisfy the needs of molecular sepsis diagnostics [8,9]. The SeptiFast (Roche) system enables the detection of more than a dozen specific microbial species, while SeptiTest (Molzym) theoretically allows to detect every possible microorganism species, but sequencing of the PCR product is required, which increases the cost and prolongs the wait for the result.

The starting point for the design of the described nested-multiplex qPCR method was the work describing the application of the qPCR method to detect bacteria and fungi in biological materials separately – Bispo et al. described the PCR methodology in the detection of bacteria with Gram differentiation in the vitreous humor, and Sugita et al. described the PCR method for the detection of yeast and filamentous fungi in the eyeball when it is inflamed [10,11]. During the work carried out by our team, it was possible to combine the sequences of primers and probes described by the authors into a multiplex reaction for simultaneous detection of bacteria and fungi with their differentiation into Gram-negative bacteria, Gram-positive bacteria, yeast fungi, and filamentous fungi. The results of sensitivity determination of such a method in the multiplex system has shown that it is possible to achieve the detection threshold of 9.9 × 10^2^ CFU/ml to 5.4 × 10^3^ CFU/ml depending on the group of microorganisms (Table 3). The resulting sensitivity was lower than the one obtained using SeptiFast (Roche) test with which one can detect the presence of individual microorganisms at the level of: 3 × 10^0^ CFU/ml for *E. coli*, 3 × 10^1^ CFU/ml for *S. aureus*, 3 × 10^1^ CFU/ml for *C. albicans* and 3 × 10^0^ CFU/ml for *A. fumigatus*[12]. In order to increase the sensitivity of the detection method in the multiplex qPCR system, a preliminary amplification procedure (I) was designed so as to gain an opportunity to carry out detection of the presence of bacteria and fungi in the nested multiplex qPCR system. The designed primer sequences and amplification procedure related to their use allowed to reduce the detection threshold to approximately 10^1^ CFU/ml for all of the four examined groups of microorganisms (Table 3). The resulting sensitivity is slightly lower than in the case of SeptiFast (Roche) test, but it should be taken into account that the number of cells of bacteria and fungi amplified in the PCR reaction oscillate at a maximum of 7.8 CFU/reaction, suggesting getting closer to the detection limit for the established conditions of nested-multiplex qPCR amplification (Table 3). Furthermore, in the SeptiFast (Roche) system, internal transcribed spacer (ITS) was used as a target region for differentiating species of bacteria and fungi, and not the sequences of 16S rRNA and 18S rRNA as in the nested multiplex qPCR method; consequently, it is not possible to directly compare the parameters of both methods [13].

The examination of blood samples from patients with clinical symptoms of sepsis, with the use of the developed methodology, gave a percentage of positive results of 69.6% compared to 18.6% obtained with the method of blood culture in the monitored culture system (Table 4). This is a considerable difference, which may raise the suspicion of false positive results, but which seems unlikely, given the use of negative control, that in each case gave a negative result. Specialized, universal media have been used in blood culture for BacT/ALERT® 3D system (bioMérieux) which could prevent the growth of certain microbial species . This could impact on the low percentage of positive results in the blood culture method. A large proportion of positive samples indicates high sensitivity of the nested-multiplex qPCR method in the diagnostics of microbiological agents that cause sepsis, but it should be remembered that the samples came from patients who experienced clinical signs of sepsis, so there was a high probability of bacteremia or fungemia. Similar results have been shown by Chang et al. in their study using SeptiFast (Roche) test, in which they demonstrated the presence of bacteria in 75% of blood samples [14]. On the other hand, the use of nested PCR increases the risk of contamination of samples, which may lead to a more frequent appearance of false positive results. Therefore, samples which are positive by nested PCR, but negative by culture may be tested by a third method (e.g. SeptiFast) in order to rule out contamination. The blood culture methods, even in automated systems, do not allow to obtain positive results of the culture in the majority of cases, which does not exclude sepsis in patients [15]. The detection of microorganisms in blood by multiplex qPCR and its sensitivity were significantly lower (Tables 3 and 4). Obviously, such results may suggest an occurrence of contamination while drawing the blood sample, when bacteria from the skin get into the sample. These are revealed at the same time as the relevant etiological agent of sepsis using the much more sensitive PCR method. In such a situation, it would be necessary to differentiate the amplification signal strength, to separate signals coming from the contamination.

## Conclusions

The method presented in this paper is based on a combination of the nested and multiplex PCR methods in order to obtain higher sensitivity; additionally, the method allows to extend its scope over the whole panel of bacterial and fungal microorganisms (with differentiation of Gram-negative bacteria, Gram-positive bacteria, yeast fungi, and filamentous fungi), but without typing specific species. This information is very useful to the physician when selecting the appropriate treatment before he receives the final identification from microbiological laboratory.

## Methods

### Reference microbial strains

Several strains were used in the research: bacteria – *Bacillus sp.* (ATCC 51912), *Enterobacter aerogenes* (ATCC 29009), *Enterococcus faecalis* (ATCC 33186), *Escherichia coli* (ATCC 25922), *Haemophilus influenzae* (DSM 4690), *Neisseria meningitidis* (ATCC 53414), *Proteus mirabilis* (DSM 4479), *Pseudomonas aeruginosa* (DSM 13626), *Serratia marcescens* (DSM 50904), *Staphylococcus aureus* (ATCC 33497), *Staphylococcus epidermidis* (ATCC 35983), *Staphylococcus haemolyticus* (DSM 20263), *Streptococcus agalactiae* (DSM 2134), *Streptococcus pneumoniae* (ATCC 49619), *Streptococcus pyogenes* (DSM 20565), *Streptococcus salivarius* (DSM 20617), fungi – *Aspergillus fumigatus* (ATCC 14110), *Candida albicans* (ATCC 10231), *Candida glabrata* (DSM 11950), *Candida parapsilosis* (DSM 5784), *Candida tropicalis* (ATCC 20115).

### Ethics statement and participants

The research was granted approval by the local Bioethics Committee of the Jagiellonian University (KBET/94/B/2009). Written informed consent was obtained from participants before their enrollment in the study.

### Blood samples

Blood was collected from volunteers, who had no clinical symptoms of sepsis and no inflammatory markers (CRP, OB). Additionally, 102 blood samples were taken from patients with clinical symptoms of sepsis, hospitalized in the John Paul II Hospital in Krakow. Blood samples were drawn into 2-ml Vacutainer K_3_E (BectonDickinson) test tubes.

### Blood culture

The blood culture was carried out in the John Paul II Hospital in Krakow in the Microbiology Department using BacT/ALERT® 3D apparatus (bioMérieux).

### DNA extraction of bacterial and fungal isolates

The bacterial and fungal DNA was isolated with the application of a specialized kit for DNA extraction (Genomic Mini, DNA Gdansk). The isolation was carried out in accordance with the manufacturer’s report.

### The method for microbial DNA isolation from blood

With the aim of determining the sensitivity of the PCR method, microbial DNA was isolated from 1.5-ml blood samples, collected from volunteers, which were simultaneously inoculated with four model microbial reference strains (*E. coli, S. aureus, C. albicans, A. fumigatus*) in order to obtain a gradient of their number from 10^5^ CFU/ml to 10^0^ CFU/ml for each one of them. DNA isolation was carried out according to the method described by Gosiewski et al. with the employment of a ready-to-use Blood Mini (A&A Biotechnology) kit [4]. The same method was used to isolate DNA from blood samples of patients with clinical symptoms of sepsis.

### DNA purity and concentration

The concentration and purity of total DNA isolates in the samples were measured spectrophotometrically at wavelengths of *A*_260_ and *A*_280_. It was performed in a NanoDrop machine (Thermo Scientific).

### DNA amplification

All the processes of DNA amplification were performed with the use of the real-time PCR method (qPCR) in a CFX96 thermal cycler (BioRad) by employing species-specific starters and TaqMan probes. The sequences of oligonucleotides utilized in the research and amplification procedures are presented in Table 1. Compositions of the reaction mixtures and the thermal amplification profiles were given in Table 2.

In each amplification reaction was used DNA isolated from the sterile human blood samples derived from healthy volunteers was used, serving as a PCR negative control.

Additionally, in every sample of DNA isolated from blood, β-actin gene detection was performed in order to check whether qPCR inhibition takes place; SYBR®Green JumpStart Taq ReadyMix (Sigma) was used for that purpose [16] (Table 1).

### Primers design

16S rDNA and 18S rDNA sequences of the following organisms were obtained from GenBank (http://www.ncbi.nlm.nih.gov/blast) provided in the public domain by the National Center for Biotechnology: bacteria – *Bacillus thuringiensis* (KC153529), *Enterobacter aerogenes* (AB844449), *Enterococcus faecalis* (KC150142), *Escherichia sp.* (KF453959), *Haemophilus influenzae* (AB377170), *Neisseria meningitidis* (AJ239312), *Proteus mirabilis* (KC150143), *Pseudomonas sp.* (JQ613981), *Serratia marcescens* (KC130920), *Staphylococcus aureus* (CP000736.1), *Staphylococcus epidermidis* (CP000029), *Staphylococcus haemolyticus* (EF522132), *Stenotrophomonas maltophilia* (AB008509), *Streptococcus agalactiae* (AB002480), *Streptococcus pneumoniae* (CP000410.1), *Streptococcus pyogenes* (AB002521), *Streptococcus salivarius* (NR042776); fungi – *Ascomycota sp.* (JX869355), *Aspergillus fumigatus* (HQ871898), *Aspergillus sp.* (KC120773), *Candida albicans* (JN941105), *Candida glabrata* (AY083231), *Candida parapsilosis* (DQ218328), *Candida tropicalis* (EU034726), *Candida tunisiensis* (JQ612155).

The universal external primers EXT_BAC_F and EXT_BAC_R (for bacteria detection) and EXT_FUN_F and EXT_FUN_R (for fungi detection) were designed by aligning in the conservative region of 16S rDNA (bacteria) or 18S rDNA (fungi), yielding products of approximately 610 bp and 440 bp. Selected sequences were aligned with 16S rDNA and 18S rDNA regions with the use of ChromasPro ver 1.7 (Technelysium Pty Ltd) software. The designed primers were later tested using Multiple Primer Analyzer (http://www.thermoscientificbio.com/webtools/multipleprimer/) software in order to check whether they form dimers or if they hybridize with one another. The primer set and probes were described in Table 1.

### The multiplex real-time amplification PCR standardization

The standardization of the method was carried out with the use of DNA samples isolated from blood (taken from healthy volunteers) simultaneously inoculated with four model reference microbial strains (*E. coli –* Gram-negative bacterium*, S. aureus* – Gram-positive bacterium*, C. albicans* – yeast*, A. fumigatus* – filamentous fungus).

Optimization of the amplification method I was carried out separately with external primers (EXT) and the amplification method II with internal primers and TaqMan probes (Table 1). Optimization of the multiplex qPCR method was based on the selection of the appropriate concentration of magnesium ion concentration as well as determining the appropriate temperature for all the four pairs of primers and the four TaqMan probes to anneal to the DNA matrix as regards amplification I and II (Table 1). For this purpose, a series of experiments was performed that tested the listed specific gradient factors: magnesium ion concentration (1.5 mM – 16.5 mM); annealing temperature: amplification I (42°C – 52°C), amplification II (56°C – 68°C).

### Evaluation of the qPCR method sensitivity

The evaluation of the PCR method sensitivity consisted in simultaneously inoculating the blood samples taken from healthy volunteers with four reference strains (*E. coli, S. aureus, C. albicans, A. fumigatus*) in the same blood sample, so as to obtain a gradient of their number from 10^5^ CFU/ml to 10^0^ CFU/ml - as regards the resulting gradient, we prepared 5 samples for each of the points representing a specific number of microorganisms. Later, DNA was isolated with the use of the methodology described above. The indication of sensitivity was performed separately for amplification II (external primers) and in the nested system, i.e. in subsequent amplifications I and II. The obtained results were compared in Table 3.

Amplification sensitivity was defined as the relation of the C_T_ value, i.e. the number of reaction cycle in which the linear increase of the product cuts the established baseline RFU (relative fluorescence unit) (Table 3).

### Statistics

The relationship between the proportion positive from each replicate of 5 and the corresponding log concentrations of the four reference strains was examined using probit regression analysis (Gretl software ver. 1.9.4.). Using the probit model, the Nested qPCR and qPCR tests were compared. A *P* value of <0.05 was taken as statistically significant.

## Competing interests

The authors declared that they have no competing interests.

## Authors’ contributions

TG was involved in protocol development, researched literature and conceived the study and wrote the first draft of the manuscript; DJ-B – patient recruitment and data analysis; AS – DNA isolation and PCR; MBW – optimization of the DNA amplification reaction; MB – data analysis. All authors read and approved the final manuscript.
